# An improvement in the reconstruction of digestive tract after total gastrectomy: ultra-short cecum

**DOI:** 10.3389/fonc.2023.1236492

**Published:** 2023-09-01

**Authors:** Shikang Ding, Xin Yang, Yibo Li, Xiaohao Zheng, Yanyang Song, Yibin Xie

**Affiliations:** ^1^ Department of Pancreatic and Gastric Surgery, National Cancer Center/National Clinical Research Center for Cancer/Cancer Hospital, Chinese Academy of Medical Sciences and Peking Union Medical College, Beijing, China; ^2^ Department of Gastrointestinal Surgery, National Cancer Center/National Clinical Research Center for Cancer, Hebei Cancer Hospital, Chinese Academy of Medical Sciences, Langfang, China; ^3^ Department of Gastrointestinal Surgery, Yun Cheng Center Hospital, Yuncheng, China

**Keywords:** total gastrectomy, ultra-short cecum, normal cecum, reflux esophagitis, nutritional status

## Abstract

**Aim:**

This study aimed to evaluate the utility and complications of ultra-short cecum (USC) in the reconstruction of digestive tract after total gastrectomy (TG) for the alleviation of reflux esophagitis and to determine its effect on long-term nutritional status.

**Methods:**

Patients who underwent TG with USC or normal cecum (NC) at a single institution between June 2018 and December 2020 were included in this study. The inclusion and exclusion criteria were defined, and the primary endpoints were reflux esophagitis, anastomotic leakage and postoperative nutritional status. The long-term nutritional status was evaluated by the change trend of laboratory blood tests, including total protein, prealbumin, hemoglobin, and total leukocytes.

**Results:**

Totally 240 cases were included in the final analysis out of 496 patients who received TG with USC or NC. Postoperative reflux esophagitis was significantly higher in the NC group than in the USC group (24.7% versus 7.7%, *P = 0.001*), and the NC group had a higher incidence of severe esophagitis symptoms compared to the USC group (13.6% versus 0.00%, *P < 0.001*), and the incidence of anastomotic leakage in the USC group was similar to that in the NC group (9.0% versus 6.2%, *P = 0.6*). There was no significant difference in long-term nutritional status between the USC and NC groups in the two years following the surgery (*P > 0.05*).

**Conclusion:**

Ultra-short cecum after total gastrectomy should be more actively recommended due to its significant reduction in reflux esophagitis and similar incidence of anastomotic leakage and nutritional status compared with normal cecum after total gastrectomy.

## 1 Introduction

Gastric cancer is the fifth most common malignant neoplasm and the fourth leading cause of cancer-related death, causing an estimated 768793 deaths worldwide in 2020, especially in Eastern Asia, Eastern Europe, and South America, based on GLOBOCAN 2020 data (1–3). Despite advancements in multimodal therapeutic approaches, such as surgery, chemotherapy, radiotherapy, and immunotherapy, surgical resection has remained the most efficient treatment for potentially resectable gastric cancer (4). According to the latest Japanese gastric cancer treatment guidelines, the standard surgical procedure for clinically node-positive (cN +) or T2–T4a tumors is either total or distal gastrectomy (5). Among all the gastric cancer patients who received surgical resection nearly 40% underwent total gastrectomy and what’s more about 15%~30% of the patients will have sever reflux after TG (6–8), and about 12.3% of patients have poor quality of life due to reflux and some of them need to be readmitted to the hospital for treatment (9).

To reduce the high rate of postoperative reflux, surgeons designed lots of method for the reconstruction of digestive tract, such as Lahey, Jejunal Interposition pouch (JIP), Roux-en-Y, Un-cut and so on (10, 11). However, reflux rate remained high till now (6, 12). Regardless of the type of gastrectomy reconstruction procedure mentioned above, an anastomosis between the esophagus and jejunum results in a segment of free jejunal cecum. The length of the cecum is usually kept between 2-3cm as recommended by the book “Gastric Cancer: Principles and Practice” (13). However, over time, remnant jejunal cecum of some patient will enlarge, and imaging examinations have shown that it becomes longer and can lead to the development of blind-loop syndrome. Reflux in some patients may be caused by the extended cecum loop. To address this issue, we have shortened the cecum between jejunum stump and esophagojejunostomy site, and we named it as ultra-short cecum (USC). In this study, based on the comparison of two reconstructive procedures after TG, including USC and NC, we observed that USC can reduce the incidence of reflux in patients without increasing the rate of anastomotic leakage, improving the postoperative quality of life of patients.

## 2 Method

### 2.1 Patient

A total of 1660 individuals diagnosed with gastric cancer underwent gastrectomy at the Cancer Hospital Chinese Academy of Medical Sciences between June 1, 2018, and December 30, 2020. Of these patients, 496 who underwent TG were included in this study. The eligibility criteria for participation were as follows: a confirmed diagnosis of gastric middle-upper adenocarcinoma (stage II-III) through digestive endoscopy and histopathological examination; regular follow-up and blood tests every 3 months in the first 2 years after surgery at this institution. Exclusion criteria included a history of other malignant tumors; extensive abdominal cavity tumor dissemination or metastasis discovered during intraoperative exploration or having undergone palliative surgery.

### 2.2 Treatment

**Figure 1:**
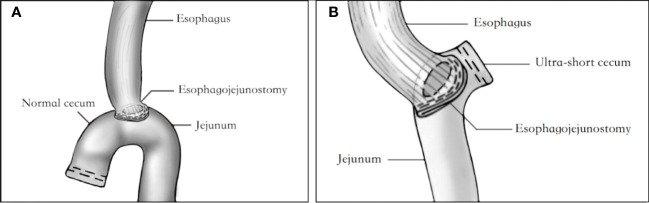
Diagram of USC and NC anastomotic reconstruction. **(A)** Diagram of USC anastomotic reconstruction. **(B)** Diagram of NC anastomotic reconstruction. USC, Ultra-short cecum; NC, Normal cecum.

The treatment methods for all patients were determined by a multidisciplinary team consisting of radiologists, pathologists, medical oncologists, and surgeons, based on the preoperative evaluation of contrast-enhanced chest-abdomen-pelvis computed tomography, upper gastrointestinal endoscopy, and other diagnostic tests. The selection of either NC or USC after TG was based on the “Japanese gastric cancer treatment guidelines (ver. 5)” and the clinical experience of the surgeons. The NC after TG involved resection of the standard D2 lymphadenectomy region, followed by reconstruction of the digestive tract using Roux-en-Y esophagojejunostomy, with a free jejunal cecum typically 2-3 cm in length (**Figure 1A**). In contrast, the free jejunal cecum side during USC digestive tract reconstruction did not usually exceed 2 cm (**Figure 1B**).

### 2.3 Study endpoint

The primary endpoints were reflux esophagitis, anastomotic leakage and postoperative nutritional status. The long-term nutritional status was assessed by measuring the percentage of laboratory blood tests that included total protein, prealbumin, hemoglobin, and total leukocyte count.

### 2.4 Data collection

Postoperative complications were classified according to the Clavien–Dindo (CD) classification of surgical complications, and any events with a grade II or higher were included in the analysis (14). Reflux symptoms were assessed using the Visick grade classification, and reflux esophagitis was diagnosed through endoscopic examination at the follow-up appointment and graded using the Los Angeles (LA) classification (15, 16). Severe reflux symptoms were defined as grade 3 or 4 on the Visick classification scale. All follow-up data, including hematological indicators at each follow-up appointment, were calculated as a percentage of the preoperative data and included in the analysis.

### 2.5 Statistical analysis

The statistical analyses were conducted using R version 4.1.5 software (www.r-project.org). Chi-square tests were used to compare categorical variables, while Student’s test for unpaired data was used for continuous variables between the two groups. A p value of less than 0.05 was considered significant. Furthermore, if the detection method varied over the years, the sva package function Combat was employed to eliminate the batch effect of laboratory items.

## 3 Result

### 3.1 Postoperative clinical and pathologic characteristics of patients

**Figure 2:**
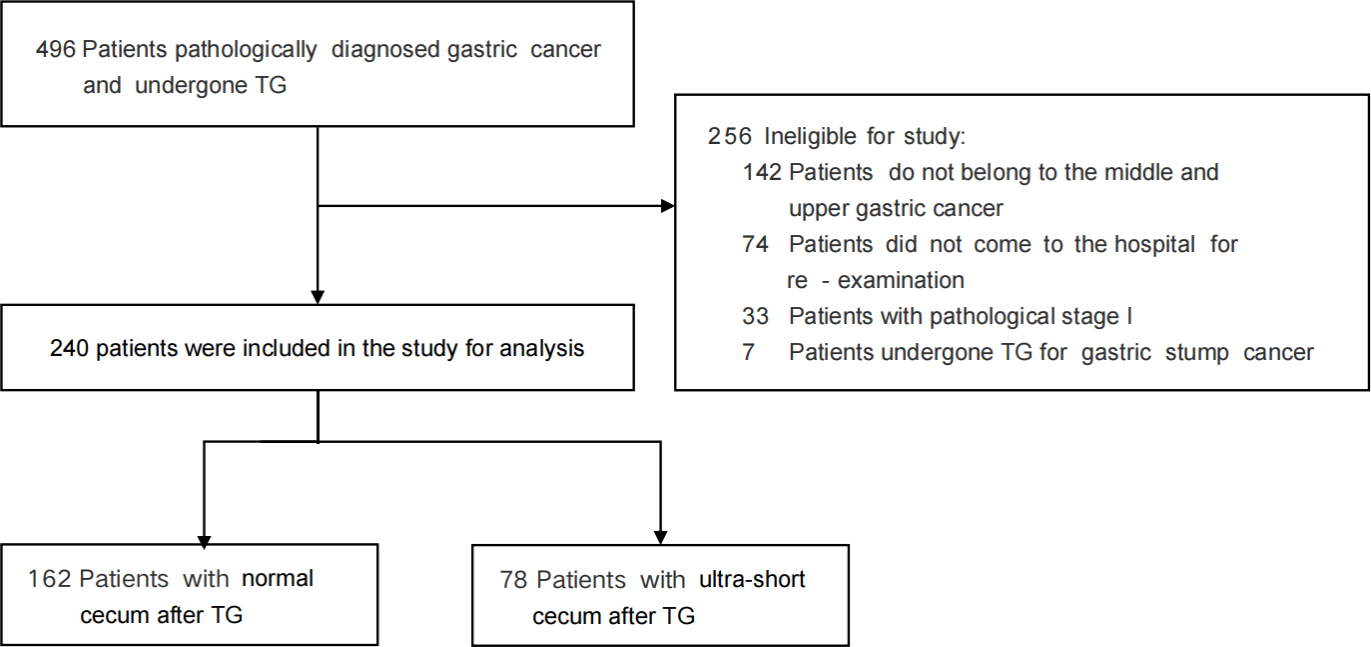
Exclusion and Inclusion Criteria.

From June 1, 2018, to December 30, 2020, a total of 240 patients diagnosed with the middle -upper gastric cancer were included in the study, with 162 in the NC group and 78 in the USC group (**Figure 2**). **Table 1** presents the clinical and pathologic characteristics of the patients. The mean (SD) age of the entire group was 57.27 (10.25) years, and 79.6% of the patients were male. There were no significant differences in preoperative demographic characteristics, including sex, age, BMI, and preoperative comorbidities, between the two groups. The operation time for the USC group was significantly longer than that for the NC group (*P < 0.05*), but the mean (SD) length of hospital stay for the NC group were significantly longer than those for the USC group (19.23 (8.21) days versus 17.06 (4.21) days, *P = 0.029*) (**Table 1**). The pathological stages for both groups were stage II or III, and the postoperative pathological characteristics were basically consistent (**Table 1**).

**Table 1:** Postoperative clinical and pathologic characteristics of patients undergoing the USC and the NC.

	USC	NC	*P*
**N**	78	162	
**Sex**			0.118
Male	57 (73.1)	134 (82.7)	
Female	21 (26.9)	28 (17.3)	0.937
**Age**	57.35 (10.85)	57.23 (9.99)	
**Comorbidities**			0.971
No	51 (65.4)	104 (64.2)	
Yes	27 (34.6)	58 (35.8)	
**BMI**	24.35 (3.01)	24.74 (3.40)	0.387
**Surgical approach**			0.303
Laparotomy	18 (23.1)	47 (29.0)	
Laparoscopy	54 (69.2)	109 (67.3)	
Laparoscopy converted to Laparotomy	6 (7.7)	6 (3.7)	
**Operation time, min, mean (SD)**	202.63 (44.40)	184.16 (63.50)	0.022
**Blooding, ml, mean (SD)**	118.59 (65.26)	124.01 (63.03)	0.538
**Neoadjuvant therapy**			0.576
No	48 (61.5)	92 (56.8)	
Yes	30 (38.5)	70 (43.2)	
**Length of hospital stay, days, mean (SD)**	17.06 (4.21)	19.23 (8.21)	0.029
**Borrmann**			0.077
Superficial	39 (50.0)	60 (37.0)	
Ulcerative	39 (50.0)	102 (63.0)	
**Differentiation**			0.114
Poorly differentiated	72 (92.3)	136 (84.0)	
Well differentiated	6 (7.7)	26 (16.0)	
**pT**			0.201
T1-T2	0 (0.0)	6 (3.7)	
T3-T4	78 (100.0)	156 (96.3)	
**pN**			0.521
N0	12 (15.4)	32 (19.8)	
N0-N3	66 (84.6)	130 (80.2)	
**pStage**			0.518
IIA	13 (16.7)	32 (19.8)	
IIB	11 (14.1)	34 (21.0)	
IIIA	21 (26.9)	38 (23.5)	
IIIB	21 (26.9)	42 (25.9)	
IIIC	12 (15.4)	16 (9.9)	
**R0 resection**	78 (100.0)	162 (100.0)	

USC, Ultra-short cecum; NC, Normal cecum.

Chi-square tests were used to compare categorical variables, while Student’s test for unpaired data was used for continuous variables between the two groups.

P ≤ 0.05 was considered statistically significant.

### 3.2 Postoperative complications

**Table 2** presents the postoperative complications. There was no statistically significant difference in the incidence of short-term complications between the USC group and the NC group, including ileus, intra-abdominal infection, pulmonary infection, hypoalbuminemia, severe eating disorder, and others (*P > 0.05*) (**Table 2**). Although the incidence of anastomotic leakage was slightly higher in the USC group than in the NC group, there was no significant difference between the two groups (9.0% versus 6.2%, *P = 0.6*). Among the long-term complications observed in 49 patients (30.2%) in the NC group and 9 patients (11.5%) in the USC group, postoperative reflux esophagitis was the most common, while there was no statistically significant difference in other complications such as ileus and anastomotic stenosis between the two groups (*P > 0.05*). (**Table 2**). **Table 3** shows in detail the incidence and severity of reflux esophagitis in the two groups, and the incidence of reflux esophagitis was considerably higher in the NC group than in the USC group (24.7% versus 7.7%, *P = 0.001*). Additionally, the Visick grade of reflux esophagitis was higher in the NC group than in the USC group (13.6% versus 0%, *P < 0.001*). Similarly, the grade C or D reflux esophagitis assessed by the LA classification was also significantly higher in the NC group than in the USC group (14.8% vs 0%, *P < 0.001*) (**Table 3**).

**Table 2:** Postoperative complications between the USC and the NC.

	NC	USC	*P*
**N, %**	162	78	
**Short complication rate**	48 (29.6)	20 (25.6)	1
** Anastomotic leakage**	10 (6.2)	7 (9.0)	0.6
** Ileus**	6 (3.7)	3 (3.8)	1
** Intra-abdominal infection**	9 (5.6)	6 (7.7)	0.722
** Duodenal stump leakage**	4 (2.5)	0 (0.0)	0.389
** Pulmonary infection**	5 (3.1)	0 (0.0)	0.278
** Hypoalbuminaemia**	23 (14.2)	10 (12.8)	0.928
** Severe eating disorder**	6 (3.7)	2 (2.6)	0.939
**Long complication rate**	49 (30.2)	9 (11.5)	0.003
** Reflux esophagitis**	40 (24.7)	6 (7.7)	0.003
** Anastomotic stenosis**	8 (4.9)	3 (3.8)	0.961
** Ileus**	10 (6.2)	3 (3.8)	0.659

USC, Ultra-short cecum; NC, Normal cecum.

Complications describe Clavien–Dindo classification grade ≥ II; and the calculation of complication rate is based on the number of individuals who experience complications divided by the total number of individuals, corresponding to the possibility that one person may experience multiple complications rather than a one-to-one relationship between each complication and a single person.

P ≤ 0.05 was considered statistically significant.

**Table 3:** Reflux Esophagitis Grading and Severity between the USC and the NC.

	NC	USC	*P*
**Reflux esophagitis**	40 (24.7)	6 (7.7)	0.003
**Severe Reflux**	22 (13.6)	0 (0.0)	0.001
**LA classification**			<0.001
A	0 (0.0)	3 (3.8)	
B	20 (12.3)	3 (3.8)	
C	20 (12.3)	0 (0.0)	
D	4 (2.5)	0 (0.0)	

USC, Ultra-short cecum; NC, Normal cecum; LA, Los Angeles.

The Los Angeles (LA) classification a clinical classification of reflux esophagitis based on endoscopic results, and the Visick classification is a symptom classification based on patient follow-up scoring; severe reflux symptoms were defined as grade 3 or 4 on the Visick classification scale.

P ≤ 0.05 was considered statistically significant.

### 3.3 Postoperative laboratory test

**Figure 3:**
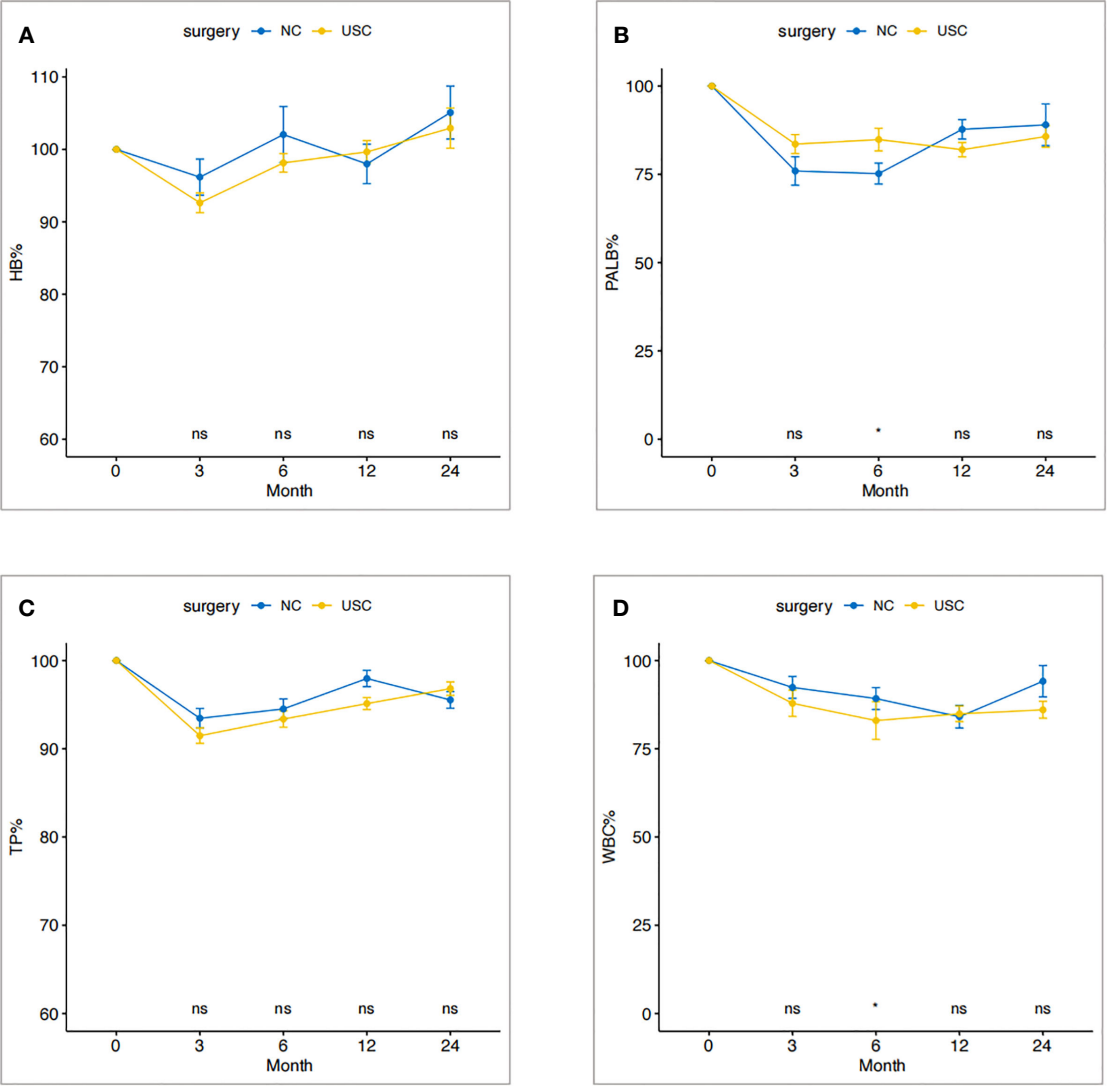
Change trend of nutritional status within two years after operation in USC group and NC group. **(A)** Hemoglobin. **(B)** Prealbumin. **(C)** Total protein. **(D)** Total leukocyte count. USC, Ultra-short cecum; NC, Normal cecum.

The trend of percentage change of laboratory nutritional indicators, including hemoglobin, prealbumin, total protein, and total leukocytes, of patients in the USC group and the NC group is shown in **Figures 3A–D**. Overall, the indicators decreased at 3 months, started to increase after 1 year, and returned to preoperative levels after 2 years. The percentage changes of nutritional indicators in the two groups were consistent at each follow-up time point (*P > 0.05*).

### 3.4 Postoperative recurrence and short-term survival

**Figure 4:**
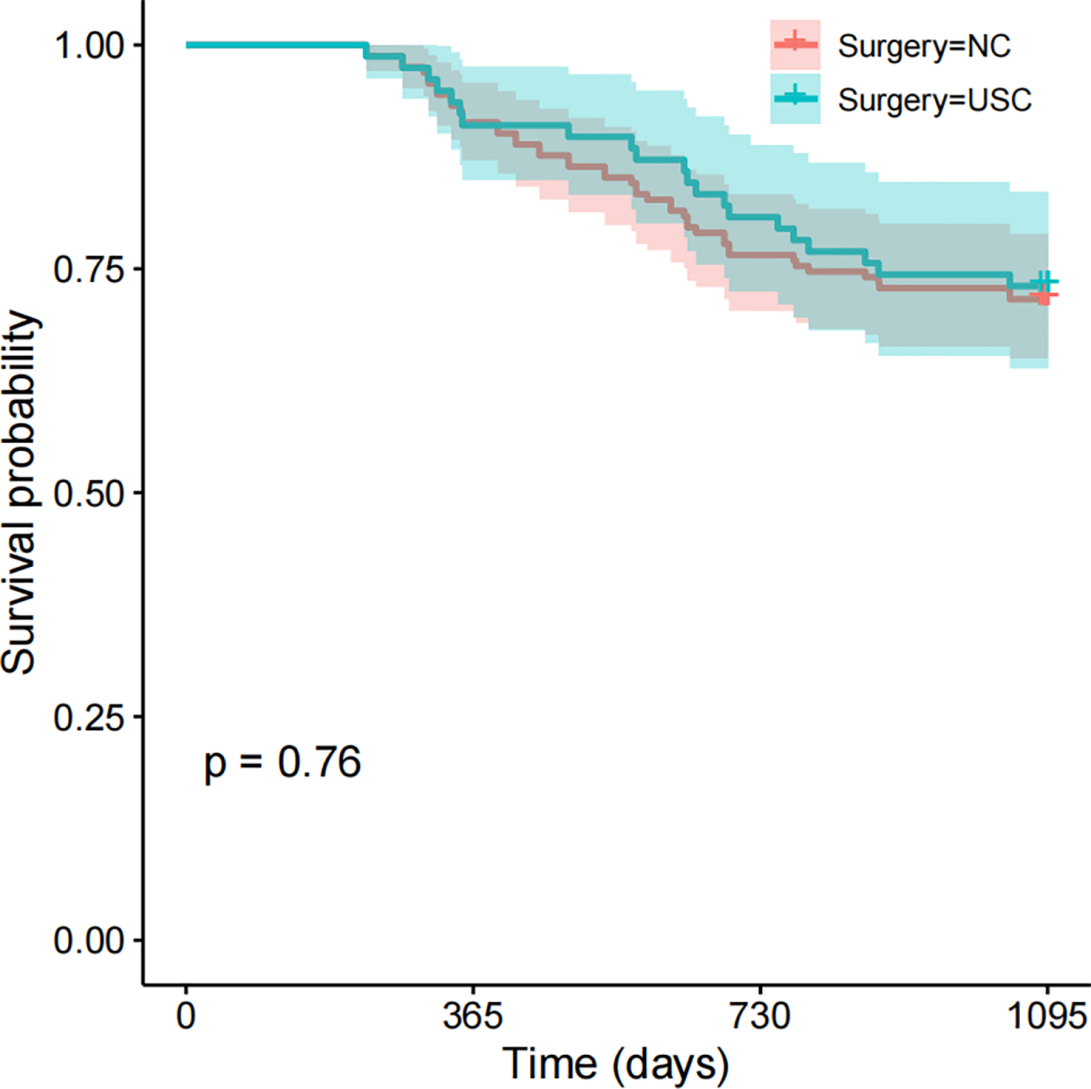
Short-term Survival between the USC group and NC group. USC, Ultra-short cecum; NC, Normal cecum.

Short-term prognosis between the USC and NC groups is shown in **Table 4** and **Figure 4**. A total of 66 patients (27.5%) experienced recurrence within 3 years after surgery, with 48 cases in the NC group and 18 cases in the USC group, and there was no significant statistical difference between the two groups (29.6% versus 23.1%, *P = 0.363*) (**Table 4**). Meanwhile, there was no statistical difference between the USC and NC groups in terms of recurrence types, including local recurrence, lymph node metastasis, hematogenous metastasis, and peritoneal dissemination (*P = 0.512*). In addition, the 3-year survival of the USC group was similar to that of the NC group (73.1% versus 71.6%, *P = 0.76*) (**Figure 4**).

**Table 4:** Recurrence Rate and Types Between the USC and the NC.

	NC	USC	*P*
	162	78	
**Recurrence**			0.363
** No**	114 (70.4)	60 (76.9)	
** Yes**	48 (29.6)	18 (23.1)	
**Recurrence type**			0.512
** Local recurrence**	2 (1.2)	0 (0.0)	
** Lymph node metastasis**	14 (8.6)	8 (10.3)	
** Hematogenous metastasis**	20 (12.3)	5 (6.4)	
** Peritoneal dissemination**	12 (7.4)	5 (6.4)	

USC, Ultra-short cecum; NC, Normal cecum.

P ≤ 0.05 was considered statistically significant.

## 4 Discussion

Gastric cancer is the second most frequently diagnosed cancer and the second leading cause of cancer‐related deaths in China (17, 18). For the middle-upper gastric cancer, TG is more suitable for larger, more invasive, and higher stage tumors, but it is associated with higher postoperative complications like reflux esophagitis, which can severely affect patient’s postoperative quality of life (11, 19–21). An early study showed that 20.90% of patients had reflux esophagitis under endoscopy despite undergoing Roux-en-Y anastomosis after TG, among whom 77.78% had Los Angeles (LA) grade C esophagitis and 5.43% of patients had concurrent reflux esophagitis and Barrett’s esophagus, which significantly affected their quality of life (6). Additionally, our previous study on esophagogastric junctional gastric cancer also indicated that 26.8% of patients developed reflux esophagitis within 5 years after TG with Roux-en-Y reconstruction (8).

To alleviate postoperative reflux, several different digestive tract reconstruction anastomosis procedures have been introduced clinically, but their effects are not ideal and have not effectively reduced the incidence of postoperative reflux (22–25). Yang et al. found that in comparison to Roux-en-Y reconstruction, patients who underwent JPI reconstruction showed differences in postoperative weight and nutritional recovery, but could not reduce postoperative reflux issues, with 16.7% of patients experiencing reflux symptoms 18 months after surgery (25). Meanwhile, other studies have indicated that although Un-cut reconstruction can alleviate the “Roux stasis syndrome” after total gastrectomy, it carries a higher risk of severe reflux disease after recanalization (24). Another procedure, the Roux-en-Y esophagojejunostomy with pouch (RPY), reduces the incidence of reflux symptoms compared to traditional Roux-en-Y anastomosis, but still reached 11.1% (7). In addition, the above reconstruction procedures can reduce the incidence of anastomotic leakage, and the free jejunal cecum is generally kept at about 3 cm. Overall, these results suggest that there is room for further improvement in digestive tract reconstruction after TG to better prevent postoperative reflux esophagitis and other complications.

In this study, we have improved a new reconstruction procedure, USC by shortening the length of the free jejunal cecum compared with the traditional esophagojejunostomy procedures. We found that USC could significantly reduce the incidence of postoperative reflux esophagitis without increasing the incidence of anastomotic leakage and other complications. The USC group had a significantly lower frequency and severity of reflux esophagitis compared to the NC group (7.7% versus 24.7%, *P = 0.003*), and there was no significant difference in anastomotic leakage between the two groups (*P = 0.6*). The length of hospital stay was significantly lower in the USC group than in the NC group (*P < 0.01*), which may be due to a slightly higher postoperative short-term complication rate in patients in the NC group than in the USC group. However, it is noted that the conclusion of this study is based on a small sample size and shorter follow-up time, and future studies need to include a large sample size and multiple centers to obtain more accurate research conclusions.

In conclusion, this study systematically explored the effects of USC and NC on various aspects of patients with the middle-upper gastric cancer, affirming that USC anastomosis can reduce the incidence of reflux esophagitis without increasing anastomotic leakage and affecting postoperative nutritional status.

## Data availability statement

The raw data supporting the conclusions of this article will be made available by the authors, without undue reservation.

## Ethics statement

This retrospective study was approved by the Ethics Committee of Cancer Institute and Hospital, Chinese Academy of Medical Sciences, and the need for informed consent was waived.

## Author contributions

SD contributed to study conception and manuscript writing and data analysis. SD, XY, YL, and XZ contributed to data collection. XZ, YX contributed to clinical treatment and diagnosis. All authors contributed to the article and approved the submitted version.
